# Learning experiences of first year graduate entry nursing students in New Zealand and Australia: a qualitative case study

**DOI:** 10.1186/s12912-023-01233-9

**Published:** 2023-03-20

**Authors:** Rhona Winnington, Kay Shannon, Rosemary Turner, Rebecca Jarden, Patricia McClunie-Trust, Virginia Jones, Eamon Merrick, Andrea Donaldson, Rachel Macdiarmid

**Affiliations:** 1 Auckland University of Technology, 90 Akoranga Drive, Northcote, Auckland, 0627 New Zealand; 2 The University of Melbourne, Parkville, VIC 3010 Australia; 3 Austin Health, Melbourne, Victoria Australia; 4 Waikato Institute of Technology, Hamilton, 3240 New Zealand; 5 University of Otago, Christchurch, 8052 New Zealand; 6 Social Science Tower, Massey University, 826 Manawatu, New Zealand

**Keywords:** Clinical placements, GEN, Graduate entry nursing programmes, Nursing students, Nursing education graduate, Practical nursing, Qualitative case study

## Abstract

**Background:**

Graduate entry nursing programmes provide students with an accelerated pathway to becoming a registered nurse. Motivations for study, together with commonly shared characteristics of students enrolling in such programmes is becoming well documented, however, their experiences of studying for a professional qualification in this manner is less understood. As a means of maintaining the relevance of these fast-tracked programmes in the future, an understanding of graduate entry nursing students’ experiences of academic teaching and clinical placements is imperative.

**Objective:**

To explore the academic and clinical experiences of students enrolled in the first year of graduate entry nursing programmes in New Zealand and Australia.

**Methods:**

A qualitative case study approach was taken. Here we report the experiences of nine students enrolled in their first year of a two-year graduate entry nursing programme during 2020. Semi-structured interviews were used for data collection and analysed using Braun and Clarke’s thematic analysis.

**Findings:**

Three overarching themes were developed—affirmation, reflections on expectations and clinical experiences.

**Conclusion:**

This study highlights the experiences of first year graduate entry nursing students, with many experiencing affirmation that their altruistic career visions came to fruition. The findings indicate that these graduate-entry nursing students interviewed for this study tended to be flexible and adaptable in their approach to study as a means of meeting the challenges of the programme, all of which are key characteristics for a registered nurse; with personal growth and the development of the self, providing preparation for their second year of study.

**Supplementary Information:**

The online version contains supplementary material available at 10.1186/s12912-023-01233-9.

## Problem

A lack of literature depicting the learning experiences of graduate entry nursing (GEN) students which is required to develop tailored programmes.

## What is already known

Graduate entry nursing students have a specific set of characteristics and learning needs that sets them apart from traditional undergraduate students.

## What this paper adds

Graduate entry nursing students are flexible and adaptable in their approach to the fast-paced programme, demonstrating maturity, personal growth, and the development of the self when clinical placements were challenging as a means of succeeding in their altruistic career choice. Furthermore, academic, and clinical components of programmes must be tailored to meet their specific learning needs.

## Introduction

Given the global nursing workforce shortage, the graduate entry nursing programmes offer one solution to building nursing capacity [1]. While much literature discusses the benefits of accelerated pathways as a means of workforce development, together with the characteristics of this student cohort [2], little is known about GEN student’s teaching and learning experiences throughout the programmes. As such, there is a clear need for an examination of GEN student’s experiences of studying for a professional qualification in this more pressured environment. This knowledge can then be used to ensure academic teaching and clinical placements are both fit for purpose and relevant, while simultaneously ensuring GEN student’s master’s level study complies with national standards set by regulatory bodies for curriculum content and minimum clinical hours – 800 h in Australia [3] and 1100 h in New Zealand (NZ) [4].

### Background

GEN programmes are well-established internationally and were introduced to NZ in 2014 [1] but have been available in the United States of America since the 1970s and Australia since the 1990s [5]. There are currently ten institutes in Australia and seven in New Zealand offering GEN programmes leading to nursing registration. The relatively small numbers of students undertaking these programmes have, however, common characteristics that set them apart from traditional undergraduate students, including maturity, life experience, a commitment to succeed [6] and high expectations [7].

The opportunity to complete an accelerated degree attracts mature adult learners [5, 8, 9] who have already been successful in their undergraduate studies [7], given the duality of gaining a higher degree alongside a professional qualification within a short time frame [7]. Commonly, students present a wide range of reasons for enrolling in a GEN programme, often relating to lack of satisfaction in previous careers, feeling drawn to the caring aspects of the nursing role and seeking an altruistic career offering professional diversity [1, 7].

While proven academic achievement contributes to self-confidence that enhances ongoing study success, some students are surprised by the complexity and difficulty of the nursing degree [7], despite their altruistic motivations to become a nurse. GEN students have narrated that they are driven to pursue a career where they can help others [1, 5, 10, 11], together with seeking fulfilment through this aspiration [12]. Current literature highlights that this mature student cohort favour active learning approaches such as a flipped classroom model, inquiry-based learning [13], or problem-based learning whereby students explore clinical cases relevant to practice [14]. As a means of contributing to the current body of nursing knowledge while simultaneously expanding the literature, this paper specifically addresses the academic and clinical learning experiences of GEN students enrolled in the first year of graduate entry nursing study. It aims to broaden our understanding of their teaching and learning needs for future programme development.

## Methodology

### Method

#### Aim

To explore the experiences of GEN students at the end of their first year of study in an accelerated nursing programme in New Zealand and Australia.

This study uses a qualitative case study approach [15] to explore the experiences of students enrolled in GEN programmes across four tertiary level education providers in NZ and Australia (three in NZ, one in Australia), after their first year of study. Ten students enrolled in the study, using a convenience sampling approach [16], and completed the first interview at the beginning of their first year of study (phase one) [1].

As this is a longitudinal and iterative study, participants will be interviewed at a further three points both during and after completion of the programme as a mechanism to understand their changing perspectives of studying on an accelerated programme. As such, phase two of the study is reported here and explores the academic and clinical experiences of students during the first 12 months of the programme. All students (*n* = ten) who participated in phase one of the study were automatically invited by email from an administrator external to the research team to avoid coercion to participate in phase two. A recruitment flyer was also placed on the learning management systems of participating universities.

#### Data collection

Nine students agreed to participate in phase two following release of all assignment results for the year. All other students were excluded. Recruitment occurred over a three-month period (December 2020 to February 2021). Participants consented and completed online questionnaires measuring demographics, date of birth, ethnicity, and gender. Individual interviews, 30–60 min in length, were arranged at a time to suit the participant and conducted virtually given the ongoing COVID-19 global pandemic. All interviews were completed by the end of February 2021. Participants received a participant information sheet and written consent form prior to interviews occurring, with written consent obtained prior to the interview. Verbal consent was confirmed at the start of each interview.

The nine students from four educational institutions consented to participate in phase two of the longitudinal study (1 was from Australia, and the remaining 8 from NZ, across 3 institutions with 4 from one and 2 from each of the others). Seven participants identified as female, two as male, and one as non-binary. The mean age of participants was 30 years (Std. Dev. 8). The youngest participant was 21 years old, and the oldest participant was 47 years old. Half of the participants described their ethnicity as New Zealand Pakeha (New Zealander of overseas descent, usually European), two participants described their ethnicity as Indian, two as other, and one as Australian European. Differences between participant demographics were not measured.

This study was conducted reflexively given the prior interaction with this participant cohort. As such, the research team’s reflection on phase one results on student motivations for enrolling in a GEN programme influenced the iterative creation of the second phase interview questions regarding student experiences while studying in their first year (Table 1). This reflexive approach highlights the research team’s active role in knowledge production [17]. Interviewers were instructed to encourage conversation from participants and when necessary to probe for deeper understandings of their experiences.

**Table 1:** Interview question schedule for phase two

Interview Schedule for Phase Two
1) Thinking back to your original motivations for enrolling in this GEN programme, tell me how studying nursing has aligned with these so far
2) Tell me what mechanisms for success you have encountered during this GEN programme of study, and what specifically has facilitated your progress last year
3) Tell me about any resources that have supported your wellbeing
4) Thinking reflectively about personal and professional growth, tell me how this GEN programme has helped you develop personally and professionally over the last 12 months
5) Looking ahead, tell me what your expectations are for the coming year in terms of study, resources you require to facilitate graduation and career opportunities?
6) Thinking about the COVID-19 pandemic and the affect it has had on you personally and as a nursing student, tell me specifically about the effects of the pandemic on your experiences—what has supported you and what has not

Four researchers external to the participants’ nursing programme, but part of the research team, undertook the interviews to reassure participants of confidentiality and impartiality on the part of the research team [18]. As a mechanism to ensure rigor in this reflexive case study, the questions were discussed and developed together as a research team, having broadly reflected on the data from phase one. An interview guide with appropriate prompts also ensure that interviewers followed the same interview structure. Interviews were audio-recorded and transcribed verbatim by either the team’s research assistant (RA) or using transcription software (checked for accuracy by the RA). All transcripts were de-identified by the RA and coded using a unique identifier to maintain anonymity of the participant.

#### Data analysis

Study data were analysed to build an understanding of students’ academic and clinical experiences during their first year in the GEN programme. Thematic analysis [17] was used to analyse the interview data, which was undertaken by three members of the research team independently in the first instance (KS, RT, RW). Utilising a, reflexive iterative approach the interview transcripts were analysed using the six-step inductive thematic analysis approach of Braun and Clarke [17], including 1) familiarising self with data, 2) generating initial codes, 3) searching for themes, 4) reviewing themes, 5) defining themes, and 6) producing the report. The COREQ checklist was also used for further rigour [19]. Text was highlighted, noting common threads and ideas that addressed the research aim. Ideas and codes were placed in a table, and them analysed and grouped according to similarity for themes. After identifying potential themes, KS, RT and RW met to discuss findings and consider the similarities and differences in interpretations of highlighted areas. Differences were explored in more detail, identifying the reasons for these differences and when interpretations differed, the transcripts were reviewed again using agreed upon codes. Following this, the patterns and themes identified were discussed and upon consensus the themes and subthemes were finalised.

#### Trustworthiness

This study has considered the four key components of trustworthiness, credibility, generalisability, dependability, and confirmability [20]. Credibility was enhanced by the triangulation of the researchers undertaking the interviews and the independent analysis of the data by other members of the research team (see previous section). In terms of generalisability, the authors have provided a clear background on the participants and the context in which they are being interviewed which will assist consumers of this research to decide on applicability to their setting. Dependability was enhanced using the COREQ checklist [19] and ensuring that the process has been documented transparently to allow replicability. Finally, the results have been reported verbatim and have been drawn directly from the data, thus ensuring the research team are not interpreting them. Furthermore, the codes and ideas were placed in a table from which themes were garnered.

### Ethical approvals

Ethical approvals and consent to participate were obtained from Auckland University of Technology Ethics Committee (19/428) and The University of Melbourne Human Ethics Advisory Group (2056175). The Auckland University of Technology approval was tabled at the Otago University Human Ethics Committee and the Wintec Human Ethics Research Group where approval was granted.

All methods were carried out in accordance with relevant guidelines and policies as per the ethical approvals obtained.

The authors can confirm that informed consent was obtained from all subjects prior to interviews occurring. Consent was written and verbal.

## Results

**Fig. 1:**
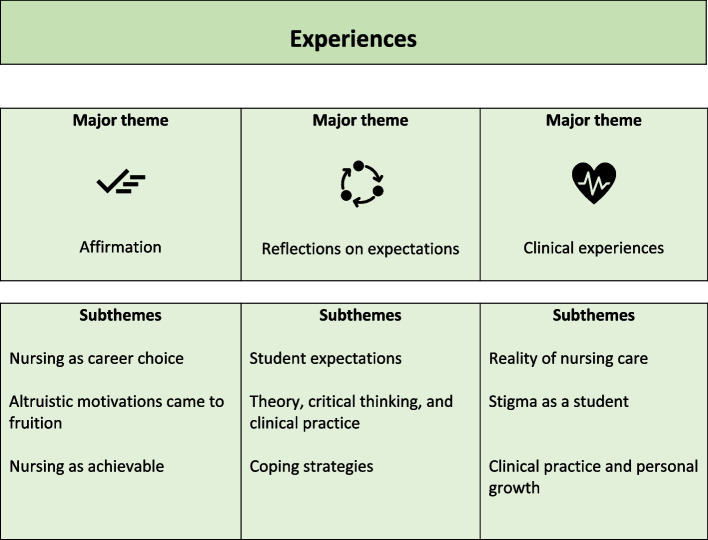
Key themes from the data

Three overarching themes were developed from the data: affirmation, reflections on expectations and clinical experiences (Fig. 1).

## Theme one: affirmation

One year into the GEN programme participants were asked to reflect on their experiences of study and whether these still aligned with their original motivations to become a RN. Overall, there was an alignment with both expectations and the reality of the programme, with three sub-themes identified from the data, these being: nursing as career choice, altruistic motivations came to fruition, and nursing as achievable.

### Nursing as career choice

The prior perception of what a nursing career would involve and the reality of experience during the first year of study for GEN students produced an alignment between expectation and reality for the most part. Students reflected on their original motivations in relation to their experiences, saying that

“all of it sounds really ridiculously romantic, but it’s like all the planets have sort of aligned. The more I’ve done it, the more I get into the programme, and it feels natural…and practice has felt natural for me” (P4).

Not all participants, however, were so positive in their statements. One participant is finding the transition to an altruistic career in nursing challenging, saying

“I thought, I’m going to leave the corporate world behind and know this real-world thing. But they’re offering you five times the salary in corporate jobs” (P7).

### Altruistic motivations came to fruition

Some participants were able to clearly articulate that their previously recorded altruistic motivations for becoming a nurse were brought to fruition during that first year of study. Their comments depicted a confirmatory journey that nursing was what they were hoping for, for example

“this is what I should be doing. It’s always felt like I should’ve just been doing this all along, and it’s a weird feeling, but it’s reassuring because it’s like, yes, this is probably it. This is me for the long run” (P4).

Furthermore, students who had some prior related experience which had prompted a shift to nursing revealed that the desire to be more in direct contact with making a difference was actualised, as

“…I did six months in research and then six months in the public health unit doing public health stuff, and it just really made me realise how much I want to be doing the face-to-face contact…I feel like I’m on the right path now…I was motivated to help people and I think it really does achieve that. So yeah, I’m really happy” (P1).

One participant gave a more specific example where the altruism came to fruition, through their story:

“…I’ve had some experiences that have been just mind blowing, there was one experience where there was a man who was very, very ill, probably didn’t have more than two weeks left to live and I was cleaning up a big mess, and he didn’t realise that I was still in the area…and I heard him speaking to someone and telling them what a huge impact I made on his life, and I was just blown away…just hearing what he was saying and thinking, wow…this is an opportunity to really make a meaningful contribution” (P7).

### Nursing as achievable

The final subtheme is demonstrated through the potential for an altruistic career that has many opportunities and longevity. Specifically, it appears that some participants had been unaware of the diversity of nursing opportunities available, as

“I hadn’t expected to find so many different areas of practice…we have had a few guest speakers and that’s brought in my understanding of what options there are for us. I had thought that I would be practicing on a ward for 20 years, but now I realise that there is so much more opportunity” (P2).

Yet, for others, their thoughts were more focused on the here and now in that time is precious when changing career, saying

“I think it was a really awesome programme, especially for someone like me who realised I wanted to do new things later on in the piece and wasn’t too keen on doing three more years…I just want to sort of get on that pathway right now” (P3).

These findings suggest that the participants in this study have embraced nursing as a career and demonstrate that their preconceived ideas about what nursing might be came to fruition; with some participants highlighting exciting experiences in their first year of study

### Theme two: reflections on expectations

Here the participants offer a clear insight into how expectations of the GEN programme did not always meet with reality, with subthemes: student expectations, theory, critical thinking, and clinical practice, and coping strategies.

### Student expectations

Participant experiences of the first year of GEN programme study were often more challenging than anticipated, and not necessarily related to participant ability but instead reflective of the programme design, as

“…the year felt very rushed, it was very cramped and there wasn’t a lot of breathing room” (P8).

Despite the reality of the GEN programme, some participants reflected on their positive experiences, saying that

“I knew I would enjoy it, but I absolutely love it. The people, the staff and the class are all wonderful” (P1), and

“it’s been a really great learning, fascinating experience…I’ve no regrets about doing it at all. Looking forward to finishing the second year and seeing where life goes” (P7).

One participant, however, reflected on their first-year experience and that more preparation would have been beneficial, suggesting that

“…some sort of preparation, even ahead of the course…that had…virtual modules or something like ‘this is what it’s like working in this kind of a health field’ or ‘this is what a night on an emergency ward could look like’…just something as a bit of preparation about what the environment is like” (P2).

This comment suggests that not all participants were aware of the realities of nursing, in a similar way to not knowing about the diversity of career options available.

### Theory, critical thinking, and clinical practice

Thinking about their experiences during the first year of the GEN programme, participants reflected on the requirements of a master’s level qualification and the ramifications of this on their personal and professional development, saying it was

“maybe a step up to a challenge and put me in positions that maybe I was a little bit uncomfortable with” (P3).

Challenges, however, were not solely about academic level, but around personal traits and thoughts influenced by life experience, for example

“I was very challenged in one of the papers to reflect on my own prejudices and ways of thinking which I think has been central to my ability to feel successful and motivated in the programme” (P2),

or challenges were presented through the delivery of programme content, in that academic staff and teaching have been

“…hit and miss. Some of them have been very helpful and then some of them not so much…I think it’s probably more like, you know some lecturers are better than others” (P4), or that

“the lecture at the time wasn’t teaching at my pace or in a way I could really engage with, so I think I just read the textbook and watched a bunch of online videos” (P6).

In relation to clinical placements, there was discord and tension as placements did not align with the theoretical content or preferences of this student cohort who, often due to their previous experiences, can be more vocal in expressing their needs, saying

“I just emailed the lecturer going…I really would like a placement which gives me exposure to ‘flare up’ [exacerbations] because, you know you’re likely to experience that in normal everyday hospital settings rather than a chronic long-term facility – I’ve landed up in a chronic long-term care facility. So, it just feels like what you’re going to get is a gamble” (P2).

Yet, there were positives also, in that participants’ learning connected both theory to clinical practice, with one commenting that the programme gives you

“…knowledge to think deeply why he’s behaving in that way rather than he’s depressed or he has depression. That’s why he’s doing that…but what’s happening inside the brain and neurotransmitters, the family, family conditions, you can think and link everything” (P5).

### Coping strategies

The accelerated pace of the GEN programme led some participants to focus on coping strategies to ensure success. Family and friends became a significant component of the coping strategies depicted, with participants saying

“I felt like quitting after the first year because it just felt so theoretical and with no experience…the support from my mother really helped me and she said it’s going to be worth it; it’s going to be worth it… [the first year] was hell, quite frankly, but like I said, it was my mother, and it was just pure encouragement” (P 1).

While personal support was essential for some participants to manage GEN programme requirements, for others their solutions were more practically focused, as

“I didn’t have any experience in academic writing, so after getting not very good marks in the first few assignments, I went to a university website on academic writing…they have a professional writing club…and they do workshops – there is always help available” (P3).

For other participants, they became the focus of their coping strategies, drawing on inner strengths, highlighting

“my inner motivation was my best resource” (P5), and.

“I’ve got all my knowledge, I know how to ask for help, I know how to ask questions” (P8).

The participants reflections focused on the programme design and the realities of being on an accelerated pathway to nursing as a career. For some they have had a positive experience, immersing themselves in study, whereas others were less complimentary regarding the organisation of the programmes.

### Theme three: clinical experiences

For many participants clinical placements were the first exposure to the realities of nursing practice, with both positive and negative experiences highlighted. Within this theme there are three subthemes reality of nursing care, stigma as a student and clinical practice and personal growth.

### Reality of nursing

For many of these participants clinical placement offers the first insight into the realities of nursing practice, with some participants sharing that the reality of nursing care was an unanticipated shock, describing their experiences as

“…the patients were very, very unwell, and I knew that hospital settings were unwell people, but it was quite shocking I think to a couple of students that I talked to” (P2),

and being surprised at

“just how long they [patients] will be in hospital and how dirty it was. So bodily fluids and so forth and how underfunded the healthcare sector was, that was the perception, not able to have the proper tubes and having to adapt and find whatever was available, and I think the lack of time with the patients, the very much treatment intervention rather than getting to know the patient – this is a different world” (P2),

or that nursing involved intimate conversations that had not been previously considered, as

“talking to people about, you know, embarrassing things or like nitty gritty things” (P3).

While these students found nursing and the conditions challenging, others were enjoying the immersion of the reality of clinical practice, highlighting that

“I think I’ve definitely learned a lot more about society’s needs, individual needs and how different everyone is, yet how similar everyone is” (P1), and

“for me…I’m definitely more comfortable talking directly with the patient and their families and things like that” (P4), and

placement was

“an opportunity to really make a meaningful contribution…I’ve had some experiences that have been just mind blowing” (P3).

Participants present a dichotomous narrative regarding their initial encounters with clinical practice as nursing students, offering a range of experiences from positive encounters to being shocked with the reality of what nursing practice entails.

### Stigma as a student

As GEN programmes recruit students who have already completed at least an undergraduate degree, they bring with them life experiences that can often be reflected upon. Specifically, they often undertake this study having already worked in other fields, thus bringing prior work and life experiences with them. This is reflected here for example

“I really had a hard kind of look at my life and thought about [what] I really enjoy [about] nursing, but I’m not sure that I would enjoy being a brand new nurse compared to going into a role with good pay, good benefits, lots of respect, lots of flexibility, better quality of life…I hated that we had that stigma or it was just [RNs] want to see you go through and earn your stripes and, you know, I’ve done that” (P7).

*“ Going into a brand new environment, especially somebody who's really pretty used to having things sorted out and then dealing with the chaos, if I'm brand new, I don't even know the layout of the building”* (P3)

*“I'm not sure that I would enjoy being a brand new nurse compared to going into a role with good pay, good benefits, lots of respect, lots of flexibility, better quality of life”* (P3)

With one of these participants finding it challenging that their life was now planned for them without consultation, saying

*“And it was, I guess, the experience being a student nurse and just being given a roster. And we don't care about your life when you are going to the bottom of the totem pole.”* (P7).

Other participants offered their thoughts about not being fully considered in terms of the clinical placements offered to them in terms of interests and locations, for example

*“sometimes it doesn’t feel like we get that much choice necessarily for our placements”* (P3).

*“you suggest and they’re like, oh, like, maybe, but, you know, any placement is a good placement. And then you're like, Yes, but I know where I want to go first week on placement”* (P4).

Participants demonstrate here how returning to student life in a professional programme produced a multi-factorial problematic narrative of having to begin at the bottom rung of the ladder given their prior life and work experience; often without consideration for their other life committments.

### Clinical practice and personal growth

The final subtheme was that of clinical practice and personal growth acquired through exposure to the realities of nursing and the possibilities it brings in achieving altruistic aims. The growth is witnessed in a number of ways, for example

“*I’m probably a more confident person because initially you have lots of interaction with strangers when you are on placements, and then you build confidence in your communication and rapport building skills. I have a broader sense of health, wellbeing within communities and people’s experiences*” (P9).

Such personal confidence is also seen when one participant explained about being a patient advocate, as

“*I would say that one of my biggest personal growths is the challenge of standing up for the patient and pushing back against situations or processes that aren’t therapeutic. For me, it’s like an internal battle, because to fit in with the norm is to ignore that ability to stand up and mention things*” (P10).

Moreover, personal growth and the development of the self is witnessed through a depiction of being challenged on biases and the realisation that despite the challenges faced during the first year, resilience and openness are essential to success, for example

“*you go in thinking that what you believe is fine and then you’re suddenly challenged on maybe the way you think, not even explicitly, but implicitly…and is like what I think actually right?…or do I have a bias against this?…so I think that’s been a big one in terms of like personal [growth]*” (P2), and

“…*there was something about this time during Covid that I found something within myself. I really revisited that place of who I am, I like that I can find that peace in the middle, in the midst of the storm, and that was something very powerful, so I was able to get through hell in my first year*” (P1).

Despite the participants previously noted desire to be part of a caring profession, these findings indicate that for some nursing was not as they anticipated. These findings indicate that there was surprise at having no voice in terms of hours worked or location of placements, as well as the shock of how unwell some service users actually are.

## Discussion

This study sought to explore the experiences of GEN students in NZ and Australia at the end of their first year of study in an accelerated nursing programme. The findings from this study highlight that for these participants their experiences in clinical practice cemented that nursing was the correct career choice for them. Specifically, participants enjoyed the realities of patient interaction during clinical placements, linking back to the altruistic motivations of these students as identified by Macdiarmid et al. [1]. Simultaneously, while enjoying making a difference for others, participants were exposed to the immense diversity of what nursing care is beyond the immediacy of the hospital ward setting and, therefore, the potential career opportunities to be had, thus aligning with the findings from Downey and Asselin [7].

In keeping with previous literature [7], the participants noted that nursing degrees, and GEN programmes specifically, are challenging, complex and require students to be dynamic in their approach to learning. Moreover, participants discussed that the expectations they had held regarding the GEN programme did not necessarily align with their experiences during the first year. Specifically, content delivery was expedited faster than anticipated leading some participants to reflect on the necessity of preparation prior to beginning study. Yet for others, this pace was met with a dynamic ability to both engage and grow as part of the process, thus aligning with Hegge and Larson’s work [21]. These experiences demonstrate that despite the demands of the programme, these participants had no regrets about undertaking the GEN programme.

The challenges experienced were, however, not solely around academic content, but encompassed prior subjective perspectives and experiences on life in general and attitudes towards these. This is unsurprising given the maturity of many of the students enrolled in GEN programmes [22] and is visible in the coping strategies used to manage the complexity of time-pressured academic learning together with clinical placement expectations [22]. Family and friends became resources of support during the first year of study particularly in relation to managing the stress related to the demands and workload of an accelerated programme, where time for hobbies and work is more limited than in undergraduate study, together with implementing health lifestyle habits thus aligning with the findings from Neill [5]. Furthermore, personal resilience due to prior life experience was certainly notable in this study, aligning with the findings of Stacey et al. [22].

This study highlights that despite the desire to have an altruistic career, a number of participants were unprepared for the reality of what nursing entailed in relation to caring for very unwell patients, the exposure to bodily fluids, and that it was hard work, thus aligning with prior research [23]. Furthermore, the need to be able to communicate with patients around personal and intimate matters was unexpected, as was the delegation of rosters without consideration of personal needs, or the burden of financial independence and need to have an income given their mature student status. Yet, despite these unanticipated experiences, participants indicated that the support of faculty and clinical educators ensured that many students evolved to embrace the nursing role, thus aligning with the work of Rico et al. [24].

For some participants the clinical exposure enhanced their holistic understanding of societies and communities through highlighting the cultural and socio-economic stratifications that exist that perhaps they had not encountered previously, either personally or professionally. As such, and similar to those described by Hegge and Larson [21], these unanticipated experiences led to enhanced individual confidence in clinical practice. Moreover, clinical experiences together with this rapid personal development produced opportunities to advocate on behalf of others, reflecting a depth of confidence that aligns with the literature in terms of GEN student profiles and common features [22]. The challenges and experiences of clinical placements for some participants proved monumental in terms of personal growth and the development of the self. Aligning with Foster et al. [25], this development of the self is notable in the GEN programme discourse and is cognisant of high achieving students’ ability to maintain wellbeing, engage actively in study and develop resilience skills, all key components of a safe and competent RN.

While this study has highlighted the experiences of some GEN students in both NZ and Australia, the findings may be relevant across many other regions globally who offer similarly structured pre-registration, GEN programmes. As such, this study depicts the experiences of these students as being similar to those detailed within the current body of literature, in that GEN programmes demand high levels of engagement from students in order to attain achievement which can be, at times, unexpected prior to commencing the programme. What comes to the fore in this study, however, is the ability of this specific student cohort to engage in the challenging academic material and clinical placements simultaneously as a means of not only being successful but also consistently high achievers in the programme [26]. As such, this commonly perceived dynamic ability to step up to challenges of this student cohort aligns closely with the work of Stacey et al. [6]. Furthermore, as a means to attain such success, GEN students clearly articulated their self-developed support mechanisms to achieve this goal.

## Conclusion

This study offers an insightful snapshot of students’ experiences in completing the first year of GEN programmes in both NZ and Australia. As such, the key findings expose a network of interrelated challenges the GEN students faced when engaging in the accelerated pathway to nursing registration. These experiences clearly highlight that exposure to the realities of nursing through clinical placements was demanding of the students but simultaneously affirmed that nursing was the right career pathway. Furthermore, this study details that despite the time pressed challenges of the programme, GEN students are able to dynamically adapt to the evolving situation through adopting prior life experiences as effective self-management tools and resilience mechanisms. Moreover, this study has clearly identified that despite the stressors of GEN programmes combined with the realities of clinical nursing placements, these students emerge from their first year of study with increasing maturity, demonstrating personal growth and the development of the self that situates them well for their final year leading to nursing registration. Despite, however, this research focusing on GEN students in both NZ and Australia, these findings may be relevant to other faculty delivering or developing GEN programmes as a means of understanding the experiences and the needs of this particular student cohort.

## Implications for research and practice

This new knowledge offers the opportunity for academic staff to ensure that future GEN programmes remain relevant in terms of teaching and learning strategies together with clinical placement experiences. Furthermore, undertaking further studies with GEN students on completion of their second year has the potential to demonstrate the ongoing growth of the nursing student self and their specific needs during an accelerated programme of study.

## Strengths/Limitations

The strength of this qualitative study is that it is part of a longitudinal case study approach, thus offering a robust methodology to access views of GEN students across the lifespan of their enrolment. This study, using a mid-point data collection technique allows the research team to develop the next stages in an iterative manner, thus ensuring alignment with actual student experiences. As such, this data offers an opportunity to inform future programme development with regard to the first year of study. A further strength is the multi-site, trans-Tasman data collection points offering multiple perspectives of these programmes. In terms of limitations, the sample size is small, however, these findings may resonate with other GEN programme providers. A further limitation of the study is that the GEN programmes included have differing numbers of both clinical and theoretical hours, however, all participants were at a similar stage of the programme at point of interview.

## Supplementary Information

**Additional file 1.**

## Data Availability

The datasets generated and/or analysed during the current study are not publicly available due privacy and ethical restrictions of the participants, but are available from the corresponding author on reasonable request.
